# Factors that influence functional ability in individuals with spinal cord injury: A cross-sectional, observational study

**DOI:** 10.4102/sajp.v71i1.235

**Published:** 2015-06-17

**Authors:** Bronwyn M. Hastings, Mokgobadibe V. Ntsiea, Steve Olorunju

**Affiliations:** 1 Department of Physiotherapy, University of the Witwatersrand, South Africa; 2 Biostatistics Unit, South African Medical Research Council, South Africa

## Abstract

**Background:**

Spinal cord injuries result in devastating impairments that can produce severe functional limitations. However, few documented studies have investigated the levels of function and factors that influence functional ability at discharge from in-patient rehabilitation facilities in Gauteng following such injuries. This necessitated further investigation.

**Method:**

Fifty participants were recruited for this cross-sectional, observational study. Participants were recruited from one private and one government spinal rehabilitation unit in Gauteng. A custom-developed questionnaire was used to establish the physical and demographic characteristics of the sample, whilst existing classification scales and measures were used to establish the degree of a lesion and a patient’s associated functional ability. Data were analysed using descriptive statistics. Multiple regression analysis was performed to determine factors that influenced the level of functional ability.

**Results:**

Patients achieved an average functional independence score of 64.6 (± 27.6) at discharge according to the Spinal Cord Independence Measure III. Longer stays at rehabilitation facilities were associated with higher scores, whereas scores decreased with increasing patient age. Pressure sores and spasticity affected scores negatively. The type of funding also influenced patients’ scores, with government funding being associated with the best outcome. Both the degree and the level at which the injury occurred could be considered predictive measures that influenced functional independence scores.

**Conclusion:**

Most participants were not functionally independent at discharge. Factors such as patient age, length of rehabilitation, presence of pressure sores or spasticity, degree of motor ability and location of the injury should be considered in tailoring rehabilitation therapy.

## Introduction

Spinal cord injury (SCI) refers to a physical injury to the spinal cord that disrupts normal spinal cord function (McKinley et al. 2001). In South Africa, SCI results mainly from motor vehicle accidents, fall from heights and violence (Draulans et al. 2011). Incidence of SCI varies depending on age, gender, region and occupation. Internationally, between 12 and 58 SCI cases are reported per million annually (Van den Berg et al. 2010).

SCIs result in devastating impairments that can cause severe functional limitations (Scivoletto et al. 2003). Able-bodied adults depend on strong arms and legs capable of well-coordinated movements and a good sense of balance when sitting, walking or moving (Van der Putten et al. 2001). Balancing upright when sitting or standing and simultaneously controlling the skilled movements of the upper limbs during a functional task, usually done automatically in able-bodied individuals, are challenging in individuals who have a SCI (Scivoletto et al. 2003).

The severity of the impairments and functional limitations depend on the extent and location of the spinal cord lesion (Itzkovich et al. 2007). Several basic skills involved in self-care activities and mobility tasks are needed for higher levels of functioning in individuals with SCIs (Itzkovich et al. 2007). Therefore, an improvement of these skills is likely to have a considerable impact on patients’ level of disability and consequently their quality of life (Van der Putten et al. 2001).

Individuals with SCIs receive in-patient rehabilitation to enhance their independence and function with their newly acquired disability. Rehabilitation that starts as soon as possible after an acute SCI has been linked with a more favourable level of functional ability (Scivoletto, Morganti & Molinari 2005; Sumida et al. 2001), and it is also advisable to begin rehabilitation as soon as possible before complications develop that can lead to secondary disability (Van der Putten et al. 2001).

Many factors may influence the functional ability of individuals with a SCI that resulted in paraplegia (Osterthun et al. 2009). Some of these factors are age at onset of the SCI, length of hospital stay (LOS), complications such as pressure sores and urinary tract infections, neurological level, gender, time to admission to rehabilitation units, and the nature of the injury (Osterthun et al. 2009).

Limited literature documents the levels of function and factors that influence functional ability following a paraplegia-causing SCI at discharge from in-patient rehabilitation facilities in Gauteng, South Africa. The rehabilitation resources and health funding models dealing with SCIs vary between clinical settings and countries. Thus, the aim of this study was to determine the levels of function and the factors that influence functional ability in individuals with paraplegia at discharge from in-patient rehabilitation facilities in Gauteng. This province was chosen as it is home to the largest share of the South African population (Statistics South Africa 2011). Knowledge of the level of function at discharge and the associated contributing factors will make therapists aware of the potential activity and participation restrictions that individuals with SCIs may face after being discharged and therefore help them determine whether specific intervention protocols need to be introduced.

## Method

### Study design and participants

This was a cross-sectional, observational study. Participants were recruited from one private and one government spinal rehabilitation unit in Gauteng, South Africa. All patients who fitted the inclusion criteria and who were nearing discharge from rehabilitation during the period of data collection (April 2011 – January 2012) were approached for possible inclusion in the study. The sample size was calculated based on the most common factors that may affect the functional outcome of persons with SCIs. The following factors were identified from literature:

- age at onset of injury
- level of injury
- time to admission after the injury
- presence of a pressure sore
- presence of a urinary tract infection.

For every factor that is considered to have a possibility of influencing the results of the study, at least ten participants are required (Nunnaly 1978). Therefore, the minimum sample size for this study was calculated as 50 participants.

To be considered for participation, individuals had to have either a traumatic or a non-traumatic SCI resulting in paraplegia, as defined by the American Spinal Cord Injury Association (ASIA) classifications A and B, and had to be older than 18 years (for consent purposes). Individuals with impairments not related to the SCI, unstable vital signs and pre-existing physical and cognitive problems were excluded from participation.

### Instruments and outcome measures

A three-part questionnaire was developed to establish the physical and demographic characteristics of the participant at discharge from an in-patient rehabilitation unit. The first section focused on demographic details and was filed separately and coded to ensure the participant’s anonymity. The second section related to possible demographic and physical factors that could influence the function of each participant, such as date of injury, date of discharge from the acute hospital, date of admission to and discharge from the rehabilitation facility, respectively, neurological injury level, age at onset of injury, gender, and set bladder and bowel routines. The third section was used to determine whether participants experienced any secondary complications since their injury, including those experienced during their hospital stay (e.g. pressure sores, urinary tract infection, pain, contractures or limited range of movement, and uncontrolled movements or spasticity of the lower limbs), reasons for early or delayed discharge, and type of health care funding (medical aid/government/workers’ compensation/private). To ensure content validity of the questionnaire, therapists with experience in the field of SCI rehabilitation and research were consulted to develop and determine whether the questions were appropriate for the aim of the study.

The ASIA classification scale of neurological impairment was used to describe the level and completeness of the lesion. Very good levels of inter- and intrarater agreement have been established in all components of the ASIA neurological examination when assessing patients with acute to chronic SCIs (Savic et al. 2007). The spinal lesions were classified according to Maynard et al. (1997) using the Oxford muscle grading scale (0–5):

- A – a complete lesion; no sensory or motor function is preserved below the level of the lesion
- B – an incomplete sensory lesion, including segments S4–S5, but no motor function below the level of the lesion
- C – sensory and motor function are incomplete, with more than half of the 10 pairs of key muscles having strength of less than grade 3
- D – sensory and motor function are incomplete, but at least half of the key muscles have strength greater or equal to grade 3
- E – sensory and motor function are normal.

The Spinal Cord Independence Measure III (SCIM III) was used to determine the level of functional ability post SCI. This is an efficient measure for the functional assessment of individuals with SCI and the concurrent validity of the scale is supported by the high correlation with the functional independence measure, although the SCIM has been found to be more responsive to change in function of persons with SCI (Itzkovich et al. 2007). The SCIM III has three subscales:

- self-care, which includes six tasks and makes up 20 points
- respiratory and sphincter management, which includes four tasks and makes up 40 points
- mobility, which is further separated into room/toilet and indoors/outdoors, which makes up 40 points.

The higher the score, the higher the level of functional independence; however, an individual with a total score below 70 is not considered to be functionally independent (Bluvshtein et al. 2012). The target SCIM scores set for high-level (T2–T9) and low-level (T10–L2) SCIs are 70.3 and 76.9, respectively (Aidinoff et al. 2011). (A low-level injury can also be considered between T10 and S5 [Long & Lawton 1955]). The main difference between the two groups of injury is that with low-level injuries, innervation of all the chest wall and abdominal muscles is retained. As abdominal muscles are used for balance and trunk stability, such injuries positively influence the ability to perform activity of daily living tasks and physical mobility skills.

### Procedure

Patients who fitted the inclusion criteria and were preparing for discharge from in-patient rehabilitation at the time were invited to participate in the study. A file was opened for each participant and the first section of the questionnaire was recorded and stored until the participant was about to be discharged. At discharge from the in-patient rehabilitation facility, the second and third sections of the questionnaire, the ASIA assessment form and the SCIM III score sheet were completed.

Using each participant’s medical file from the hospital, the researcher completed the questionnaire with the participant and assessed the participant according to the ASIA scale. Each participant was observed performing the tasks detailed in the SCIM III score sheet within the rehabilitation unit. All 50 trials included in this measure were conducted by the same researcher and in the same sequence with every participant. The researcher gave instructions to each participant before a trial and recorded all the data during each trial. Participants were not allowed to watch one another completing the SCIM III tasks to minimise participant learning, which could affect the reliability of the tests. The ASIA scale indicating each participant’s neurological level and the SCIM III score indicating the participant’s level of functional ability were also recorded during this session.

### Ethical considerations

Ethical approval for this study was granted by the University of the Witwatersrand Ethics Committee on Human Research (M10810).

### Data analysis

Data were analysed using descriptive statistics for age, gender, classification of funder, time to rehabilitation admission, LOS, ASIA motor score, all post SCI complications, and SCIM III score. A univariate analysis was conducted on all items using STATA 10 statistical software to determine any significant factors that affected the functional ability of SCI patients at discharge. This was followed by a multiple regression analysis on only those factors with a moderate significance (*P* < 0.1). However, data were not distributed normally; therefore, the data were transformed to achieve normality before analysis. After the univariate analysis was performed, a stepwise regression was performed. Variance inflation factors (VIFs) were checked for possible co-linearity. None of the VIFs were close to 10 and only factors with VIFs above 10 would warrant further investigation for co-linearity; therefore, there was no effect of co-linearity in the results.

## Results

A total of 50 patients participated in this study. Average age, LOS, time to rehabilitation admission and ASIA motor scores are shown in Table 1. The average age at the time of SCI was 40 (± 15.35) years. There were more male participants (80%, *n* = 40) than female participants (20%, *n* = 10). Most of the participants in this study sample qualified for workers’ compensation, which means that they had an injury on duty (40%, *n* = 20), followed by those who used medical aid (26%, *n* = 13), received government-funded treatment (22%, *n* = 11) and those who paid privately for their rehabilitation (12%, *n* = 6).

**TABLE 1:** Descriptive statistics as determined for the study sample (*n* = 50).

Factor	Mean	Standard deviation	Minimum	Maximum
Age (years)	40	15.35	18	80
Time to admission to rehabilitation (days)	168.03	10.00	7	1640
Length of rehabilitation stay (days)	89.56	25.84	12	216
ASIA motor score	42	7.76	20	50

ASIA, American Spinal Cord Injury Association.

Participants’ general physical characteristics are shown in Table 2. The majority of participants suffered traumatic injuries (76%, *n* = 38), sustained at a low spinal level (68%, *n* = 34), and followed either a set bladder management programme (82%, *n* = 41) or a bowel management programme (78%; *n* = 39).

**TABLE 2:** Physical characteristics of the study sample (*n* = 50).

Physical characteristic	Number of individuals	Percentage of sample (%)
Cause of injury		
Traumatic	38	76
Non-traumatic	12	24
Level of injury		
High-level injury (T2–T9)	16	32
Low-level injury (T10–S5)	34	68
TLSO used in rehabilitation		
Yes	8	16
No	42	84
Bladder management regime		
Yes	41	82
No	9	18
Bowel management regime		
Yes	39	78
No	11	22
Readmission to acute hospital during rehabilitation		
Yes	2	4
No	48	96

TLSO, thoracolumbar sacral orthoses.

Post SCI complications experienced by participants are shown in Table 3. Of the total sample, 42% (*n* = 21) experienced back pain, whereas 12% (*n* = 6) experienced shoulder pain. About a third of the participants (34%, *n* = 17) developed a pressure sore during their acute hospitalisation (before being referred to a rehabilitation hospital), whereas 26% (*n* = 13) developed a pressure sore during their stay at the rehabilitation facility.

**TABLE 3:** Complications experienced after spinal cord injury in the study sample (*n* = 50).

Complication	Number of individuals	Percentage of sample (%)
Back pain		
Yes	21	42
No	29	58
Leg pain		
Yes	5	10
No	45	90
Shoulder pain		
Yes	6	12
No	44	88
Pressure sores (acute hospitalisation)		
Yes	17	34
No	33	66
Pressure sore (rehabilitation facility)		
Yes	13	26
No	37	74
Urinary tract infection		
Yes	17	34
No	33	66
Spasticity		
Yes	12	24
No	38	76
Decreased range of movement in lower limbs		
Yes	3	6
No	47	94

**FIGURE 1:**
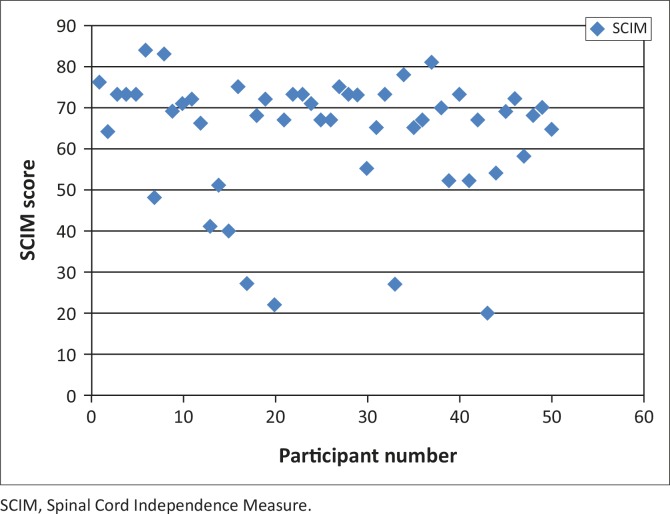
Distribution of functional ability scores as determined by the Spinal Cord Independence Measure III (*n* = 50).

Figure 1 shows the distribution of the SCIM III scores for this study sample. The average SCIM score in this sample was 64.6 (± 27.6). Participants with high-level injuries had an average score of 55.9, whereas those with low-level injuries had an average score of 67.5.

SCIM, Spinal Cord Independence Measure.

The results of the multiple regression analysis of the factors that influenced functional ability of individuals post SCI are presented in Table 4. For every one year increase in the age of the participant, the SCIM score dropped by about 0.18%. For every day by which LOS increased, the SCIM score increased by 0.06%. SCIM scores of participants who developed pressure sores were 9% lower than those of participants who did not develop pressure sores. Similarly, SCIM scores of participants who experienced spasticity were 8% lower than those of participants without spasticity.

**TABLE 4:** Factors that had a statistically significant influence on functional ability scores of individuals with spinal cord injuries at discharge from in-patient rehabilitation.

Factor	Coef.	Std. err.	t-value	P > |t|	95% Conf. interval
Age	-0.18	0.09	-2.07	0.05	-0.397
LOS	0.06	0.02	2.34	0.03	0.01–0.1
Pressure sore at acute hospital	-9	3.36	-2.68	0.01	-15.8 – (-2.18)
Spasticity	-8.34	3	-2.78	0.01	-14.4 – (-2.3)
Classification of funder†	-	-	-	-	-
Workers’ compensation	-4.82	3.35	-1.44	0.16	-13.62
Medical aid	-8.07	3.64	-2.21	0.03	-15.5 – (-0.7)
Private	-10.84	4.07	-2.67	0.01	-19.1 – (-2.6)
Level of injury‡	-	-	-	-	-
ASIA motor score	1.29	0.21	6.23	< 0.001	0.87–1.7
Low (T10–S5)	6.64	0.05	-2.12	0.01	0.76–0.96

ASIA, American Spinal Cord Injury Association; Coef., coefficient; Conf. interval, confidence interval; LOS, length of hospital stay; Std. err., standard error.

Reference: †, Government; ‡, High (T2–T9).

Relative to participants whose SCI rehabilitation was government funded, individuals whose therapy was funded by workers’ compensation (owing to being injured on duty) scored 4.8% lower on the SCIM III score sheet, followed by participants who used medical aid or private insurance (8.1% lower SCIM scores) and participants who paid out of their pockets for rehabilitation (10.8% lower SCIM scores). Participants with low-level injuries had a 6.6% higher SCIM score relative to those with high-level injuries (*P* = 0.01).

## Discussion

The average SCIM score in this study was 64.6 at the time of discharge from in-patient rehabilitation. The majority of the participants in this study were therefore discharged from rehabilitation without having reached functional independence. Although higher scores represent higher functionality, an individual with a total score below 70 is not considered to be functionally independent (Bluvshtein et al. 2012). Similar results were found in a study by Aidinoff et al. (2011), who found that the average SCIM score at discharge for individuals with SCI-related paraplegia was 67.8, close to the score reported in the current study. Wirth et al. (2008) assessed the functional ability of 64 patients with complete motor paraplegia both on admission to and at discharge from a rehabilitation hospital, and again one year post injury. They found that the mean SCIM score increased from 60 at discharge after 3 months (earliest discharge) to 71 at discharge after 6 months (latest discharge time). A similar trend was observed in the current study, with individuals discharged after an average hospital stay of approximately 3 months not being functionally independent. This may be an indication that patients needed further rehabilitation, as our analysis showed that for every additional day spent in rehabilitation, the SCIM score increased by 0.06%. A longer hospitalisation may contribute to a higher cost of rehabilitation, which may have influenced some patients’ decision to opt for continued rehabilitation on an out-patient basis; however, this was not specifically established in this study.

The functional independence at discharge was also influenced by the level of injury. In this study, participants with high-level injuries had an average SCIM score of 55.9, whereas those with low-level injuries had a score of 67.5. Aidinoff et al. (2011) found that individuals with complete SCIs consistently achieved higher SCIM scores at discharge from rehabilitation when the neurological level of injury was more caudal. However, in our study neither patients with high-level injuries nor those with low-level injuries achieved scores close to the targets set by Aidinoff *et al*. and therefore are not considered to have achieved functional independence relative to their level of injury. These differences may be due to differences in the methodologies and demographics of the study populations, as the average age of participants in our study was higher and the average LOS was shorter than in the comparative study of Aidinoff *et al.*

Increased age and a shorter LOS may influence functional ability at discharge. Our results showed that for every one year increase in the age of the participant, the SCIM score dropped by about 0.18%. This was an expected result, as it has been reported previously that in individuals with paraplegia, age appears to adversely affect functional outcome (Cifu et al. 1999; McKinley, Cifu & Seel 2003; New 2007). New (2007) also found a statistically significant negative correlation between the participant’s age and the mean score on the motor subscale of the functional independence measure at discharge. This may be because older patients with SCIs may have reduced functional reserves and greater comorbidities compared with younger patients, and are more likely to have had physical impairments prior to their SCI (McKinley et al. 2003).

Our results showed that for every additional day spent in rehabilitation, an increase of 0.06% in the SCIM score could be expected. This was an expected result as other studies have similarly found that a longer hospital stay is associated with a higher functional gain (McKinley, Tewksbury & Mutjteba 2002; Ronen et al. 2004). The longer the in-patient rehabilitation period, the greater the opportunity for an individual with a SCI to achieve most of their functional goals before discharge (Ronen et al. 2004).

Individuals who developed pressure sores achieved 9% lower SCIM scores than those who did not develop pressure sores. In this study, 36% of participants developed a pressure sore during their acute hospitalisation and 26% developed a pressure sore whilst at the rehabilitation facility. During rehabilitation, the maintenance of an existing pressure sore or the management of a new pressure sore requires the individual to avoid any pressure to that area until the wound is fully healed. For example, an individual with a sacral pressure sore will not be allowed to sit or lie supine until the wound is fully healed. This management approach may delay the individual’s functional ability as it leads to avoidance of functional positions, prolonged periods of immobility, longer hospitalisation, depletion of health care funding for further rehabilitation, delay in the overall accomplishment of rehabilitation goals and a delay in the acquisition of wheelchair skills (Chen, DeVivo & Jackson 2005).

For participants with spasticity, SCIM scores were 8% lower compared with those of patients who did not experience spasticity. Yelnik et al. (2010) also reported that spasticity may contribute to decreased functional ability post SCI. Moderate spasticity may allow individuals with lower limb paresis to perform all mobility transfers with more ease and independence, therefore allowing higher overall SCIM scores to be achieved (Adams & Hicks 2005). However, severe spasticity may contribute to decreased functional ability, contractures, incorrect posture, pressure sores and pain, all of which may negatively affect an individual’s mobility and ability to perform activities of daily living tasks independently (Yelnik et al. 2010).

SCIM scores of participants with low-level injuries were 6.6% higher than those of participants with high-level injuries. As noted earlier, a similar outcome was seen in individuals with complete SCIs as determined with the functional independence measure (Aidinoff et al. 2011). Similar results were also reported by Bluvshtein et al. (2012), who indicated that an individual’s functional ability is determined by the level of spinal cord injury. The lower the level of injury, the more muscles that are fully innervated and the better an individual’s strength and balance. This makes it easier for the individual to accomplish physical mobility tasks and functional goals than when the injury is at a high level (Bluvshtein et al. 2012). This is supported by our findings using the ASIA motor score, which showed that for every unit increase in the ASIA motor score, there was an increase of 1.3% in the SCIM score.

Relative to participants whose rehabilitation was government funded, SCIM scores of individuals who were injured on duty and therefore received workers’ compensation were 4.8% lower, followed by participants who used medical aid or private insurance (8.1%) and participants who paid out of their pockets for rehabilitation (10.8%). Similar trends could not be found reported in literature, but from the authors’ clinical experience, individuals whose rehabilitation expenses are covered by government tend to have a relatively longer hospital stay than those who use medical aid or private insurance. Should a health care funder not supply adequate funds for full rehabilitation in a private rehabilitation facility, individuals using medical aid may be discharged prematurely. Owing to medical aids increasingly curbing expenses, patients are not eligible for prolonged stays in rehabilitation units (Ronen et al. 2004), which may negatively affect the functional ability of an individual with SCI (McKinley et al. 2002).

Government-funded patients achieved relatively better functional ability at discharge from rehabilitation, which may be due to their relatively longer hospitalisation (these patients usually have an opportunity to stay in a rehabilitation unit until their in-patient rehabilitation goals are met). However, there is often a shortage of government-funded SCI rehabilitation beds (Hardcastle 2011). Some individuals go to private SCI rehabilitation units, but often do not have adequate funds to pay for prolonged rehabilitation and subsequently they do not experience the full benefit of rehabilitation.

### Limitations

Although this study identified factors that may influence the functional ability of individuals at discharge from rehabilitation, it did not focus on the functional ability of the participants on admission into rehabilitation, psychosocial factors, the type and duration of daily therapy sessions or the level and intensity of spasticity. However, despite these limitations, the findings of the study provide useful insights for clinical practice.

## Conclusion

The majority of the participants in this study were discharged from in-patient rehabilitation facilities before they could reach functional independence. It may therefore be necessary to emphasise the importance of outpatient rehabilitation after discharge or consider increasing the LOS to help them reach optimal functional independence. Factors such as an advanced age, short rehabilitation stays, the presence of pressure sores or spasticity, low ASIA motor scores and high-level SCIs may warrant close monitoring for functional outcomes and more attention during in-patient rehabilitation. When working towards functional recovery of an individual with a SCI, therapists should do regular ASIA testing to monitor changes in an individual’s neurological level of injury and take into account the functional implications of the level of the lesion.
